# The Global Self-Reactivity Profile of the Natural Antibody Repertoire Is Largely Independent of Germline D_H_ Sequence

**DOI:** 10.3389/fimmu.2016.00296

**Published:** 2016-08-10

**Authors:** Andre M. Vale, Cecília B. Cavazzoni, Alberto Nobrega, Harry W. Schroeder

**Affiliations:** ^1^Department of Medicine, University of Alabama at Birmingham, Birmingham, AL, USA; ^2^Department of Microbiology, University of Alabama at Birmingham, Birmingham, AL, USA; ^3^Department of Genetics, University of Alabama at Birmingham, Birmingham, AL, USA; ^4^Program in Immunobiology, Laboratory of Immunereceptors and Signaling, Carlos Chagas Filho Institute of Biophysics, Federal University of Rio de Janeiro, Rio de Janeiro, Brazil; ^5^Department of Immunology, Paulo de Goes Institute of Microbiology, Federal University of Rio de Janeiro, Rio de Janeiro, Brazil

**Keywords:** natural antibodies, self-antigen recognition, CDR-H3 repertoire, peritoneal B cell subsets

## Abstract

Natural antibodies (NAbs) are produced in the absence of exogenous antigenic stimulation and circulate in the blood of normal, healthy individuals. These antibodies have been shown to provide one of the first lines of defense against both bacterial and viral pathogens. Conservation of the NAb repertoire reactivity profile is observed both within and across species. One view holds that this conservation of NAb self-reactivities reflects the use of germline antibody sequence, whereas the opposing view holds that the self-reactivities reflect selection driven by key conserved self-antigens. In mice, B-1a B cells are a major source of NAbs. A significant fraction of the B-1a antibody repertoire is devoid of N nucleotides in H chain complementarity determining region 3 (CDR-H3) and, thus, completely germline encoded. To test the role of germline D_H_ sequence on the self-reactivity profile of the NAb repertoire, we examined the composition and self-antigen specificity of NAbs produced by a panel of D_H_ gene-targeted BALB/c mice, each strain of which expresses a polyclonal, altered CDR-H3 repertoire that differs from the wild-type norm. We found that in most cases the same key self-antigens were recognized by the NAbs created by each D_H_-altered strain. The differences in reactivity appeared to represent the genetic signature of the NAb repertoire of each mouse strain. These findings suggest that although germline CDR-H3 sequence may facilitate the production of certain NAbs, a core set of self-antigens are likely the main force driving the selection of Nab self-specificities.

## Introduction

“Natural antibodies” (NAbs) are immunoglobulins present in the healthy organism in the absence of intentional immunization (1). The stimuli behind the production of NAbs by B cells have been a topic of considerable interest since NAbs were first recognized. Several studies have shown that germ-free (GF) and even antigen-free (AgF) mice produce serum IgM in amounts equivalent to mice raised under normal vivarium conditions, suggesting that NAbs are largely independent of stimulation by external antigens, including antigens derived from the microbiota (2–4). Instead there is considerable support for NAb production being driven by endogenous antigen stimulation (5). Self-antigen recognition appears to play a physiological role in the distribution of B cells within subpopulations, often into distinct anatomical niches (6–8).

B-1a lymphocytes are generated preferentially early in ontogeny and have been implicated as a main source of NAbs. The B-1a cells demonstrate decreased or absent N-region addition at the V-D and D-J junctions and are thus, enriched for entirely germline-encoded Ig variable domains (9–17). Given that B-1a cells are among the first B cells to develop in the fetus, their participation in the secretion of NAbs might provide a reasonable explanation for the conservation of the Nab repertoire and its establishment early in ontogeny (18, 19). However, studies with irradiated chimeras reconstituted with fetal liver and adult bone marrow from wild-type (WT) mice have shown that most NAb reactivities are produced even when B-1a are profoundly reduced in numbers, thus suggesting a role for somatic selection in regenerating the NAb repertoire of specificities (20).

Although previous studies have shown that the reactivity profile of NAbs remains conserved regardless of gut colonization or depletion of B-1 cell compartment (2, 3, 20); the influence of inherited CDR-H3 antigen-binding sites on the NAb repertoire remains unclear. BALB/c mice IgH loci contain 13 functional D_H_ gene segments, each belonging to one of four families (DFL, DSP, DST, and DQ52). Theoretically, each D_H_ gene segment gives the developing B cell access to six reading frames (RFs) of differing peptide sequence. In practice, however, the use of RF1 is preferred, RF2 and RF3 are used less frequently, and the three inverted RF are rarely used (21). We had previously used techniques of cre–loxP-based gene targeting to delete 12 of the 13 D_H_ gene segments in the BALB/c D_H_ locus, retaining only the single DFL16.1 segment (ΔD-DFL mice) (22). We then generated the ΔD-iD strain by replacing the center of the single DFL16.1 segment with an inverted DSP2.2 gene segment (23). D_H_ gene-targeted BALB/c mice express polyclonal, altered CDR-H3 repertoires that differ from the WT norm [reviewed in Ref. (24)].

We have previously observed that the ΔD-iD strain lacks NAbs that protect against infection with *Streptococcus pneumoniae* while continuing to exhibit protective reactivity against a key altered self-antigen, oxidized LDL (25). In the present work, we sought to investigate, in depth, the nature and extent of the conservation of NAb reactivities with self-antigens from brain tissue. To test the extent to which germline antibody gene content controls the composition of NAbs and the innate antibody response to self-antigens, we examined the NAb repertoire in a panel of WT and D_H_ gene-targeted BALB/c mice raised under specific pathogen-free conditions. We found that most, but not all, of the reactivities against self-antigens were found in the serum of mice irrespective of changes in germline D_H_ content and, thus, CDR-H3 sequence and structure. Our findings suggest that for most self-reactivities the primary force driving the generation of NAbs is exposure to a key set of self-antigens.

## Results

### Conservation of Serum IgM Reactivity Profile (Actual Repertoire) in D_H_-Altered Mice

**Figure 1:**
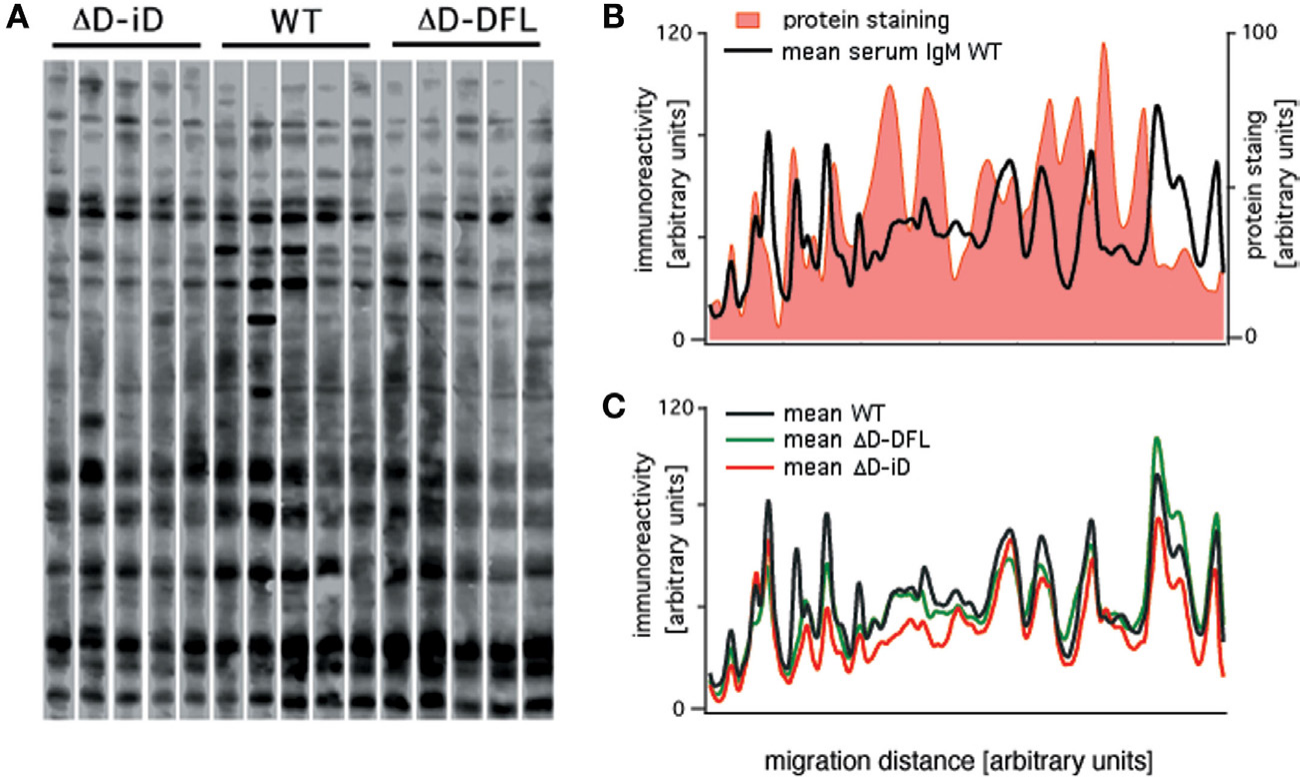
**Serum natural IgM pattern of self-reactivity expressed by ΔD-DFL, ΔD-iD, and WT BALB/c mice**. **(A)** Serum IgM reactivities against syngeneic brain extract from five animals of each strain. **(B)** Total protein staining by colloidal gold densitometric profile was compared to natural IgM recognition profile in the same membrane. **(C)** Mean serum IgM reactivity profiles of each mouse strain. Raw data adapted from Ref. (25).

Serum IgM repertoire of antigen specificities have been screened using a semi-quantitative immunoblot assay that enables accurate *en bloc* analysis of the self-reactivity of IgM present in the serum (*actual repertoire*) of unmanipulated mice (26–28). We used the same assay to analyze the serum IgM reactivities of D_H_-altered mice with self-antigens from brain tissue. Shown in Figure 1A is a comparison of the pattern of self-reactivity expressed by the WT BALB/c, ΔD-DFL, and ΔD-iD strains. To test whether the reactivity levels correlate with immunoglobulin specificity or with the relative abundance of the proteins, we compared protein staining with immunoreactivity densities in the same membrane. The comparison shown in Figure 1B reveals highly abundant proteins that are not recognized by natural serum IgM, as well as highly IgM-recognized proteins that are not abundant in the tissue extract. With a few notable exceptions, the mean sera IgM reactivity profiles were remarkably conserved among mouse lineages that generate very different primary CDR-H3 repertoires (22, 23) (Figure 1C).

It is noteworthy that these data revealing restriction of self-antigen recognition comply with the idea of the *immunological homunculus*, as proposed by Cohen (29). Furthermore, these data demonstrate that the reactivities found in the immunoblot assay cannot be attributed to simple non-specific interactions.

### Impact of D_H_ Alterations on the Available Repertoire

The results shown above suggest that strong mechanisms of antigen-driven somatic selection may operate at the cellular level to recreate a normal WT NAb repertoire of specificities in genetically modified mice. In mice, the peritoneal cavity (PerC) is the major site for both B-1 and B-2 cells. We, thus, sought to investigate whether these somatic selection pressures would generate similar repertoires among the PerC B-1 and B-2 B cells (available repertoire) from the different mouse strains. The reactivity profiles of these B cell populations provided a privileged opportunity for comparison between different B cell populations that share the same anatomical niche. After sorting each PerC B cell population (B-1a, B-1b, and B-2), we cultured the cells under LPS stimulation to induce polyclonal IgM secretion and tested the supernatants using the immunoblot against brain extract assay.

**Figure 2:**
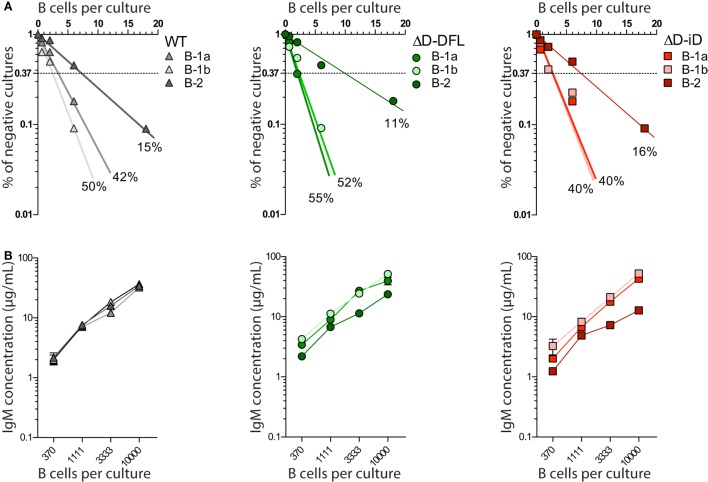
**D_H_-altered B cells response to polyclonal stimuli**. **(A)** Frequencies of response to LPS of PerC B cell subsets of each mouse strain were calculated by limiting dilution analysis. Presence of IgM in culture supernatants of 18, 6, 2, and 0.66 cells/culture on day 7 was assessed by ELISA. **(B)** IgM production by each PerC B cell subset in culture was measured by ELISA on culture supernatants of increasing number of cells/culture (370, 1111, 3333, and 10,000 cells/culture). PerC B-1a cells were sorted as B220^lo^CD5^+^, B-1b as B220^lo^CD5^−^ Mac-1^lo/+^, and PerC B-2 cells were sorted as B220^hi^CD5^−^Mac-1^−^. All cultures received 30 μg/mL of LPS and 5 × 10^3^ S17 feeder cells. Supernatants were collected on day 7.

All three subsets from D_H_-altered PerC B cells demonstrated frequencies of response to LPS that were similar to their corresponding WT B cell subsets (Figure 2A). Production of IgM in culture was also similar (Figure 2B). Thus, D_H_ alteration and restriction of the CDR-H3 repertoire did not affect the B cell response to polyclonal stimulus from LPS.

**Figure 3:**
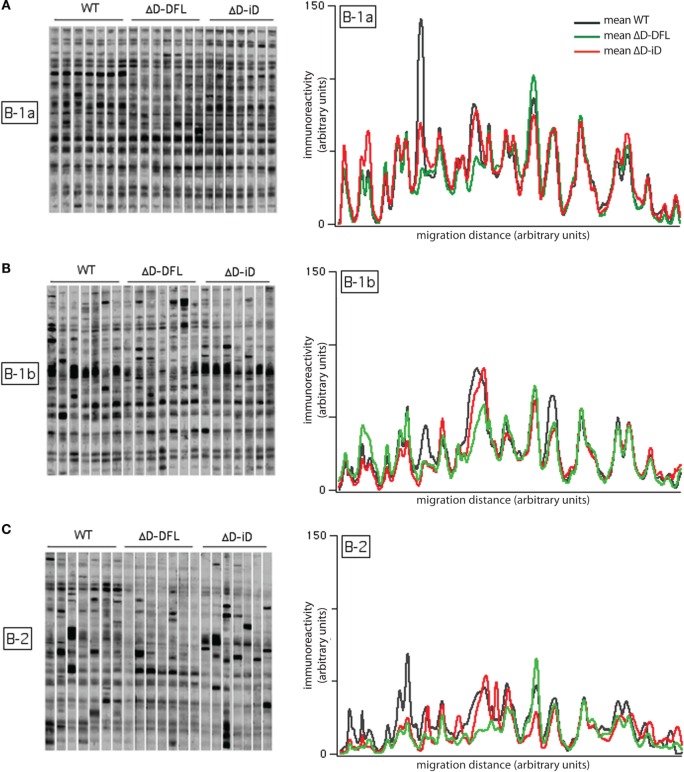
**Self-reactivities of the available repertoire in PerC B cell subsets**. Left panels **(A–C)** show 21 B-1a, B-1b, and B-2 cells culture supernatants (~1,500 responding B cell per culture), respectively, reacting with syngeneic brain extract. Right panels show the mean intensity of reactivities displayed by B-1a **(A)**, B-1b **(B)**, and B-2 **(C)** cells from each mouse genotype. Raw data adapted from Ref. (25).

We then performed the same analysis of self-reactivities shown for serum IgM with the supernatants from the B-1 and B-2 cultures (Figure 3). As there is a difference in the frequency of response to LPS among B cell subsets, we diluted each supernatant to normalize all to the same IgM concentration per growing clone (30). We performed an immunoblot analysis of the reactivities against syngeneic brain extract from seven separate culture supernatants from the three strains of mice, for a total of 21 supernatants for each B cell subset.

The mean intensity of reactivities present in B-1a cells culture supernatants remained almost completely invariant despite the genetic alteration of the D_H_ gene sequences (Figure 3A, right). The mean reactivity profiles for B-1b cells supernatants (Figure 3B, right) also demonstrated similarities in self-antigen reactivities irrespective of genotype, although the replicates in the membrane suggested a greater degree of variation around the mean values (Figure 3B, left). Conversely, the mean reactivity profiles of the B-2 cells were divergent both within and across the different mouse strains. This corresponded well with the different sets of reactivities of the B-2 cells supernatants (Figure 3C, right). The individual variability of immunoreactivities of the 21 B-2 cell culture supernatants were also highly increased when compared to the B-1 reactivity profiles (Figure 3C, left). These findings suggested that differences in the variance of the reactivities were intrinsic to the B cell subset, and not dependent on the germline sequence of the immunoglobulin repertoire.

**Figure 4:**
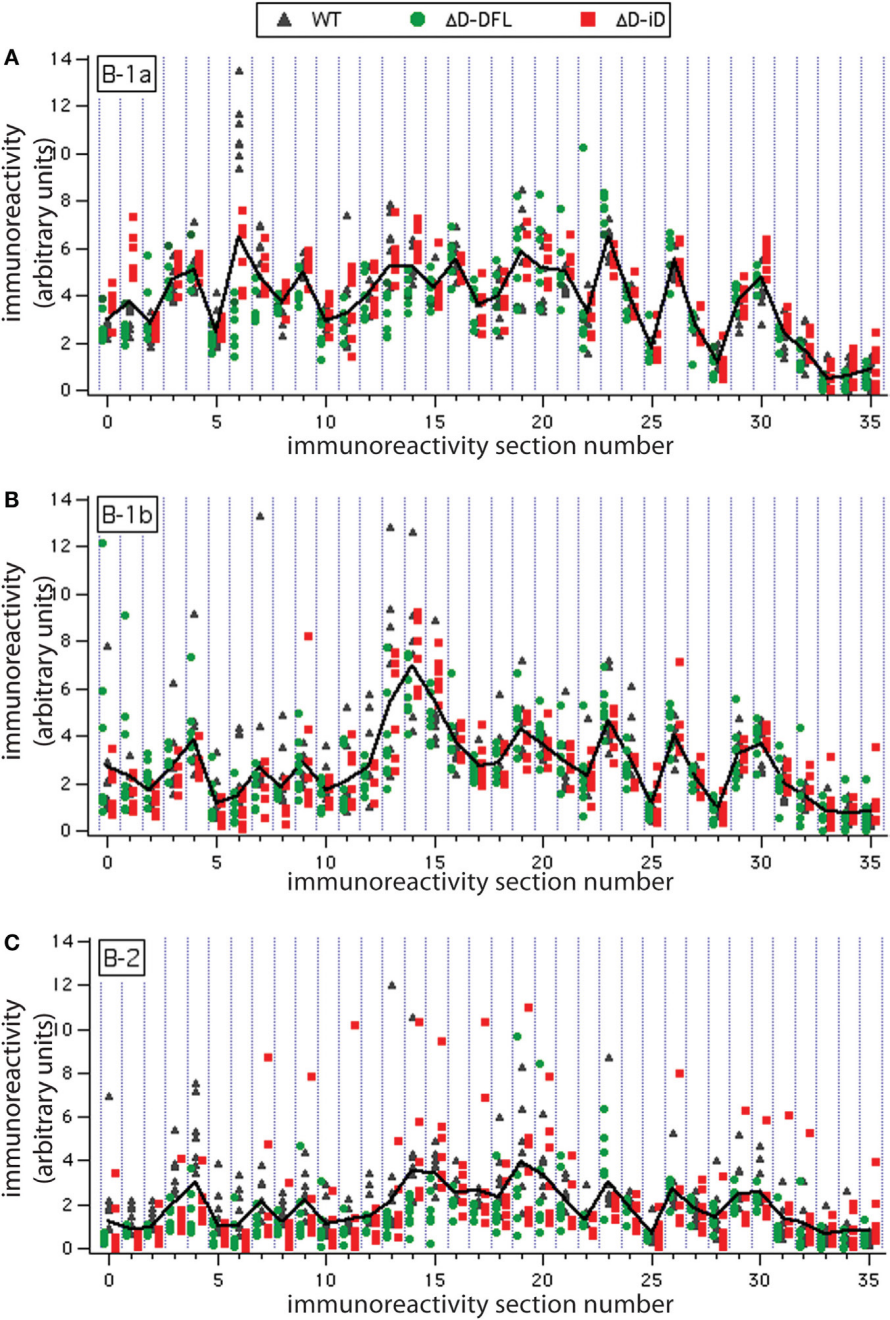
**Variance distribution of reactivities around the mean densitometric profile for each PerC B cell subset**. Supernatant magnitudes of reactivity against the 36 sections in which reactivity profiles divided are plotted in **(A–C)**. These corresponded to B-1a, B-1b, and B-2 subsets, respectively. Solid lines indicate the mean profile of all supernatants for each B cell subset.

To address the issue of variance in depth, we divided the immunoreactivity profiles into 36 sections, which corresponded to the major bands of reactivity. We plotted the magnitudes of reactivity against these sections for each supernatant from the B-1a, B-1b, and B-2 subsets (Figures 4A–C, respectively). The solid lines indicate the mean profile of all supernatants for each B cell subset. In this format, the variance of the distribution around the mean profile can be properly visualized. Despite few exceptions, we observed that the variance around the mean was much lower for the B-1a supernatants when compared to B-1b and B-2 (Figure 4).

### Each PerC B Cell Subset Displays a Characteristic Reactivity Repertoire Signature

**Figure 5:**
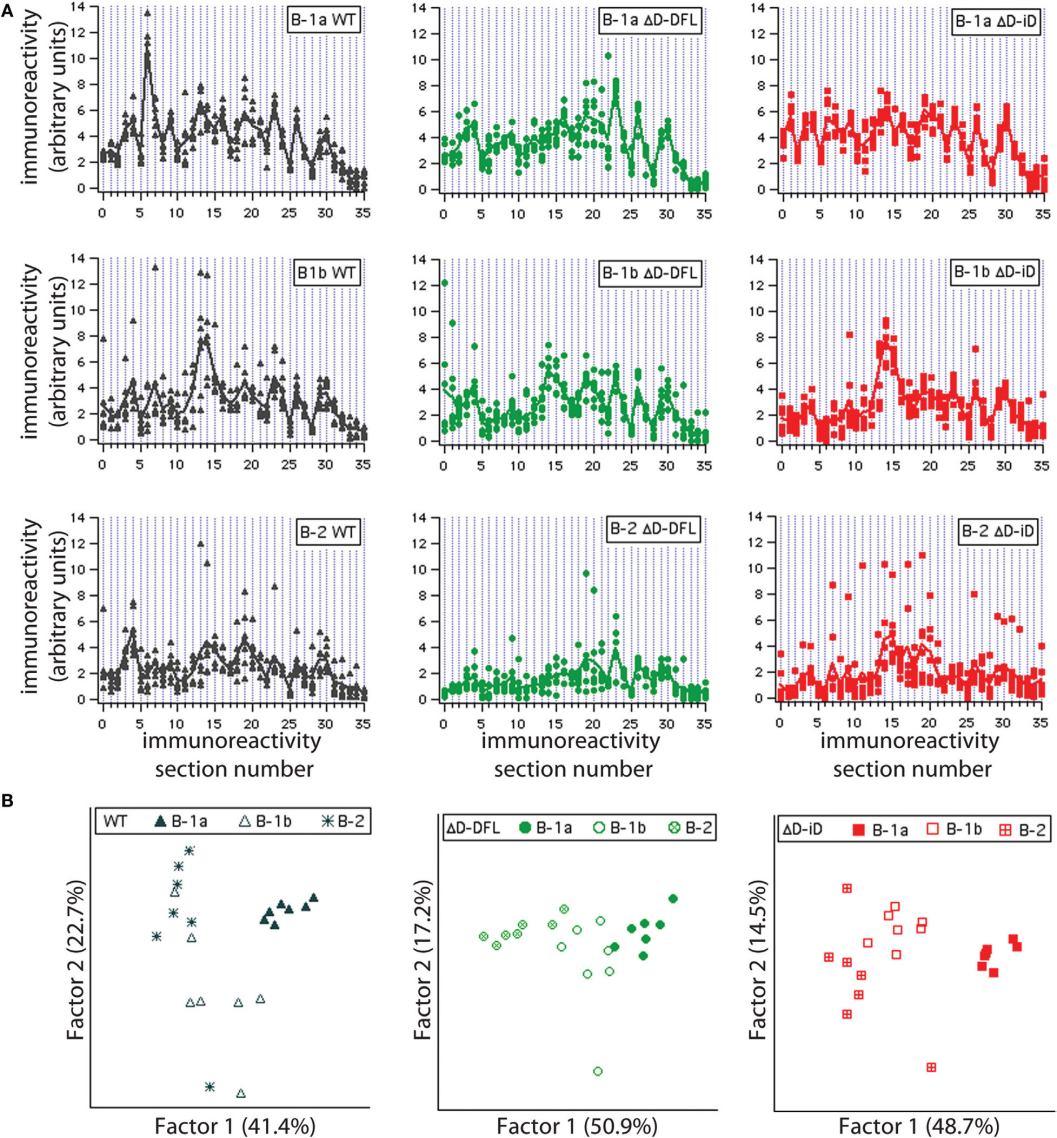
**Graphic representation of variance distribution of each B cell subset reactivity profile within each mouse strain**. **(A)** Profiles of reactivities and distribution around the mean profile of each PerC B cell subset from WT, ΔD-DFL, and ΔD-iD are represented graphically. **(B)** Principal component analysis (PCA) of reactivity profiles from each mouse strain discriminates B cell subsets.

To further confirm that each B cell subset displays a unique repertoire, we analyzed the reactivity profiles within each mouse strain. Irrespective of D_H_ content, the B-1a individual culture supernatants profiles proved very homogeneous, with little variance around their mean (Figure 5A, top; and Figure S1 in Supplementary Material). The reactivity profiles from B-1b subset were observed to differ from the B-1a for all mouse strains (Figure 5A, middle). The B-2 reactivity profiles displayed increased variance of the distribution in WT mice and especially for the ΔD-iD strain (Figure S1 in Supplementary Material), whereas the single normal D_H_ control (ΔD-DFL) displayed less variability and intensity of reactivity (Figure 5A, bottom).

To substantiate these observations, we performed principal component analysis (PCA) for each mouse strain. This multivariate approach takes in consideration all the reactivities together, providing an unbiased evaluation of the data (26, 31). All PerC B cell subset repertoires were discriminated within each mouse strain, suggesting distinct repertoire signatures for each population.

### The Conservation of the Self-Reactivity Profile Correlates with B Cell Subset in Preference to Germline D_H_ Sequence Content

**Figure 6:**
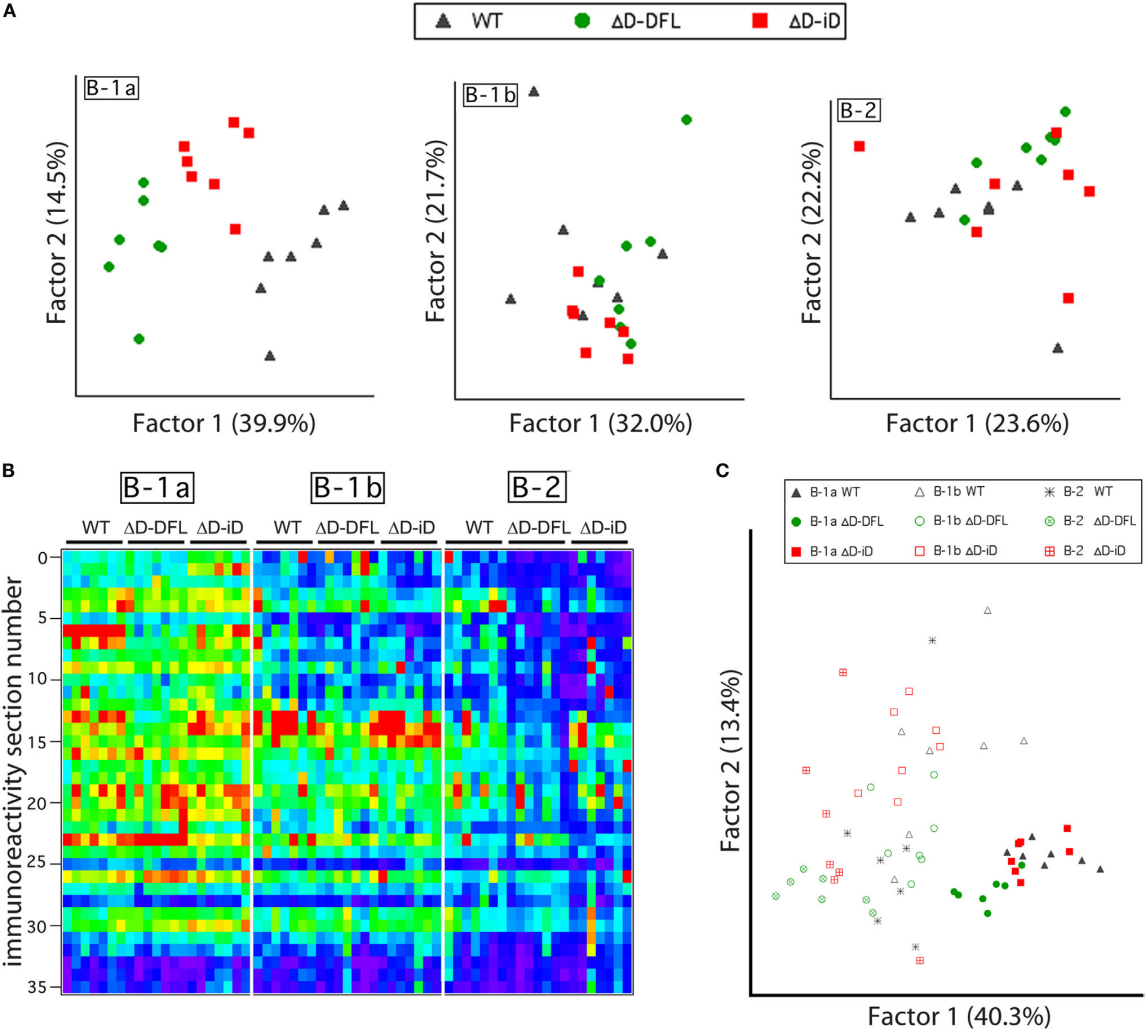
**B cell subset-intrinsic and genotype-dependent features of the repertoire**. **(A)** Principal component analysis (PCA) of reactivities for each PerC B cell subset discriminates the B-1a genetic alterations of D_H_ sequences in the available repertoire of specificities but not B-1b or B-2 subsets available repertoire. **(B)** All data from reactivity profiles (B-1a, B-1b, and B-2 subsets from the three mouse strains) are shown on a single heat map indicating similarities between reactivity profiles. **(C)** Subjecting all data matrix to PCA results in subset characteristics prevailing over genetic distinctions imposed by D_H_ alterations.

Although the mean reactivity profiles were generally conserved among the three mouse strains (Figure 3A), individual differences between the repertoires were also apparent (Figures 2 and 3). We, thus, performed a PCA of the data presented in Figure 4, which are grouped by B cell subset. This multivariate analysis revealed the impact of the genetic alteration of D_H_ sequences on the B-1a available repertoire of specificities (Figure 6A, left), demonstrating distinct differences between the strains. To better evaluate the contribution of each factor to the resulting B cell subset repertoires, we collected all the data from the reactivity profiles (B-1a, B-1b, and B-2 subsets from the three mouse strains) on a single heat map (Figure 6B). This large data matrix was similarly subjected to PCA.

Both the immunoblot assay (Figures 2 and 3) and the heat map of the reactivities (Figure 6B) disclosed a set of major bands that were unique to each strain and were shared among individual mice from each strain. These shared similarities and differences were much less apparent in the B-1b and B-2 subsets (Figure 6A). When analyzed by PCA together, the characteristics common to the subsets appeared to exert a greater effect than the characteristics provided by the differences in D_H_ content (Figure 6C).

## Discussion

Natural antibodies are formed early in ontogeny and are, thus, mostly germline encoded (18, 19). We sought to test whether the specificity of the NAb repertoire is the product of natural selection of germline immunoglobulin sequence or whether self-antigen is the driving force. These two hypotheses are not mutually exclusive, because even if self-antigen were the stimuli, the use of conserved germline sequence could facilitate the development of antibodies with certain specific epitope recognition properties that could provide protection in an anticipatory fashion (25).

In this work, we focused on the role of the sequence encoded by CDR-H3, which is created *de novo* by VDJ rearrangement and N addition and lies at the center of the antigen-binding site. This central position allows CDR-H3 to often play a major role in defining the epitope specificity of the antibody (32). The diversity gene segment (D_H_) contributes significantly to the amino acid composition of CDR-H3. The ΔD-DFL strain expresses that portion of the CDR-H3 repertoire that is typically generated through use of the DFL16.1 gene segment, and is thus enriched for tyrosine, serine, and glycine. The ΔD-iD strain forces use of amino acids contributed by DSP gene segment inverted sequence. This repertoire is enriched for arginine, histidine, and asparagine.

Even though the CDR-H3 repertoire differs between the three strains of mice, the global self-antigen specificity of serum IgM in all three strains was largely the same (Figure 1), suggesting that antigen reactivity, and not germline immunoglobulin sequence content, was the driving force. The immunoreactivity profiles were similar in mice missing that portion of the immunoglobulin repertoire that is normally created by the 12 other deleted D_H_ gene segments in the ΔD-DFL mice. The same finding was obtained in the ΔD-iD mice that are not only missing the contribution of 12 D_H_ gene segments but also preferentially use an inverted RF sequence that completely alters the normal contribution of D_H_ encoded amino acids to the repertoire.

In previous studies in WT BALB/c mice, we found evidence of categorical selection of CDR-H3 sequence during B cell development, with B cell subsets, including those from the PerC, often exhibiting characteristic global sequence signatures (15, 33–35). These signatures were in addition to the bias for germline sequence exhibited by B-1a. To test whether the IgM repertoire expressed by these subsets in our panel of mice varied in its self-antigen specificities, we sorted B-1a, B-1b, and B-2 cells from the PerC, cultured them in the presence of LPS, and examined the IgM that was produced (Figure 3). If the self-specificity of the IgM were to reflect the influence of germline sequence, then the prediction was that B-1a IgM expressed by the D-altered B cells would demonstrate the greatest deviation from the WT norm. However, the opposite was observed. The reactivity of the IgM produced was most conserved in B-1a with the least variance, and least conserved in B-2 with the most variance (Figures 3 and 4). The B-1a repertoire, thus, demonstrated major convergent self-antigen-binding selection specificities in the presence of divergent germline sequence, whereas the B-1b and B-2 repertoires did not. PCA confirmed the distinct nature of the repertoire produced by each of the three subsets (Figure 5). The mechanisms behind these differences in variability are unclear, but potentially could reflect B cell subset-intrinsic characteristics, such as self-renewal capacity, clonal size, and repertoire polyreactivity.

The dominance of the biology of the B-1a component of the repertoire over the genetic alteration of CDR-H3 content among the activated PerC B cells, which is revealed by the heat-map PCA (Figure 6C), is in agreement with the substantial homology of the self-reactivities of the IgM in the sera of the WT, ΔD-DFL, and ΔD-iD mice (Figure 1), which has been argued to derive primarily from the B-1a subset (16). Although the biology of each individual subset exerted the greater effect on the reactivity of the IgM produced, a multivariate analysis revealed individual differences between the repertoires as a function of D_H_ sequence (Figure 6). By inspection, it is clear that some of these individual differences reflect the presence or absence of specific bands.

ΔD-DFL mice bear a D_H_ segment that is expressed in 20–30% of B-1a WT; this may result in a large overlap of B-1a repertoires and explain the similarity of their NAb repertoires. However, this explanation does not hold for ΔD-iD mice. One possible mechanism that could explain the convergence of the reactivities would be an antigen-influenced shift in the immunoglobulin repertoire expressed by the D-altered PerC B cells in the use of V_H_ or J_H_, the extent of N nucleotide insertion or exonucleotide nibbling, or use of alternative D_H_ RFs. Studies are underway in our laboratory to test these hypotheses.

Our findings of similar immunoreactivity profiles between D-altered mice indicate that NAb reactivity is likely driven by a key subset of self-antigens. The nature of these antigens and the epitopes that define them remain largely unknown. In WT animals, they are preferentially recognized by IgM formed by germline-conserved CDR-H3 sequences. In mice forced to use alternative sequences, the majority, but not all, of these self-antigens seems to be able to induce a NAb repertoire with a similar reactivity profile. The exceptions may represent epitopes that require unique, germline-encoded D_H_ sequence in the antigen-binding site (25). These findings strongly suggest that it is the self-antigens, rather than conserved CDR-H3 germline sequence, that play the greater role in driving NAb production and reactivity.

## Materials and Methods

### Mice

The panel of D_H_-altered BALB/c mouse strains was bred in our mouse colony at the University of Alabama at Birmingham (UAB). Mice bearing ΔD-DFL (22) or ΔD-iD (23) D_H_ alleles were created by cre–loxP targeting of a BALB/c ES cell line. For each allele, 12 of the 13 BALB/c D_H_ gene segments were deleted and then the single, remaining DFL16.1 gene segment was either retained or altered. All WT and D_H_-altered mice were studied at from 8 to 10 weeks of age. The mice were maintained in a SPF barrier facility and in a climate-controlled environment with a 12 h light/12 h dark cycle, with diet and water supplied *ad libitum*. Animal care was conducted in accordance with established guidelines and protocols approved by the UAB Animal Care and Use Committee.

### Flow Cytometry and Cell Sorting

Flow cytometric analysis and cell sorting were performed as previously described (36). Briefly, peritoneal washout cells were obtained from five different mice of each D_H_-altered strain. The following mAbs were used to isolate PerC B cells into the B-1a, B-1b, and B-2 subpopulations: anti-B220 (RA 3.6B2) (BD Pharmingen) (Southern Biotechnology, Birmingham, AL, USA), anti-Mac-1 (BD Pharmingen, San Diego, CA, USA) and anti-CD5 (BD Pharmingen, San Diego, CA, USA). PerC B-1a cells were sorted as B220^lo^CD5^+^, B-1b as B220^lo^CD5^−^Mac-1^lo/+^, and PerC B-2 cells were sorted as B220^hi^CD5^−^Mac-1^−^. A MoFlo instrument (Dako) was used for cell sorting.

### Electrophoresis and Immunoblot

The preparation of brain extract, as well as determination of protein concentration, electrophoretic separation, blotting onto nitrocellulose membranes, and the test of immunoreactivities in the Mini-Cassette System, was performed as previously described (27) with secondary anti-IgM antibody coupled to alkaline phosphatase from Southern Biotechnology (Birmingham, AL, USA). The four gels used for limiting dilution assay (LDA) of reactivities were polymerized and run in parallel, such that profiles of total proteins blotted (revealed with colloidal gold) and the immunoreactivity profiles of lanes containing the standard pooled supernatant matched accurately between the gels, both qualitatively and quantitatively.

### Rescaling of the Immunoblots and Data Analysis

The densitometric profiles of immunoreactivities were analyzed as previously described (30). Briefly, profiles were acquired first by scanning (Silverscanner II). This was followed by colloidal gold staining in order to reveal the migration position of the proteins. A comparison between any two immunoreactivity profiles could subsequently be performed by referring to the corresponding protein profiles. Data analysis was performed using the software IGOR Pro (Wavemetrics, Lake Oswego, OR, USA). Special software packages were written by the authors for the analysis and statistical treatment of the data and can be obtained from the authors.

### B Cell Culture and Limiting Dilution Assay

B cell cultures were performed as described previously (36). Sorted B-1a, B-1b, and B-2 cells were cultured in 250 μl of complete RPMI medium in 96 well flat-bottom plates in the presence of 30 μg/ml of LPS (*Salmonella typhimurium*, Sigma-Aldrich). All cultures contained 5 × 10^3^ S17 feeder cells/well for growth support as described by Ref. (37) with some modifications. Briefly, 1 day before the start of the LDA cultures, 5 × 10^3^ S17 cells were added per well and incubated overnight at 37°C with 5% CO_2_. The next day, the S17 culture plates were irradiated with 3000 rad and various numbers of the sorted B cells were added to the cultures. Culture supernatants were used to determine the frequency of IgM secreting clones by ELISA according to the Poisson distribution (38, 39) and contained sorted B cells with 22 replicates for each cell number (18, 6, 2, and 0.66 B cells per well). Another culture set was performed to analyze reactivity profiles by immunoblot, with 22 replicates of 10,000; 3333; 1111; and 370 B cells added per well. Culture supernatants were typically harvested on the fifth day of culture, unless indicated otherwise in the text.

### IgM ELISA

To determine IgM concentration in the supernatants, ELISA was performed as previously described (36), using anti mouse IgM-specific reagents (Southern Biotechnology). Standard curves, obtained by using polyclonal, serially threefold diluted, mouse IgM (Southern Biotechnology), were used to quantify IgM.

### Statistical Analysis

Differences between populations were assessed by Student’s *t*-test, two tailed; Fisher’s exact test, two tailed; χ2-test; or Levene’s tests for the homogeneity of variance, as appropriate. Analysis was performed with JMP version 7.0 (SAS Institute, Inc., Cary, NC, USA). Means are reported with the SE of the mean.

## Author Contributions

AV, AN, and HS designed research; AV performed the experiments; AV, CC, and AN performed the statistical analysis; AV, CC, AN, and HS analyzed data and wrote the manuscript.

## Conflict of Interest Statement

The authors declare that the research was conducted in the absence of any commercial or financial relationships that could be construed as a potential conflict of interest.

## Supplementary Material

Figure S1 **The immunoblot reactivity profiles of culture supernatants from LPS-stimulated B cells were subdivided into 36 reactivity sections, as shown in** Figure 4. For each mouse lineage and each B cell subpopulation, seven different supernatants were tested in the immunoblot assay for immunoreactivity against self-antigens derived from brain tissue extract (Figure 4). The variance of the magnitude of immunoreactivity for all 36 sections of reactivity was calculated for each mouse lineage and each B cell population. Histograms are shown in the figure. In all cases, most of the reactivity sections presented variance values below 10^6^ units. For comparison between histograms, the number of sections with variance values inferior, or superior, to 10^6^ units was indicated for each case. The differences between histograms were tested for statistical significance with Fischer exact test applied to 2 × 2 table containing the numbers of reactivity sections with variance values inferior, or superior, to 10^6^ units.

## Abbreviations

ΔD-DFL, depleted D_H_ locus with a single DFL16.1 gene segment; ΔD-iD, depleted D_H_ locus with a single mutated DFL16.1 gene segment containing inverted DSP2.2 sequence; CDR-H3, complementarity determining region 3 of the immunoglobulin heavy chain; LDA, limiting dilution assay; NAb, natural antibody; PCA, principal components analysis; PerC, peritoneal cavity; TdT, terminal deoxynucleotidyl transferase; WT, wild type.
