# Supplementary figures and images for: The Global Self-Reactivity Profile of the Natural Antibody Repertoire Is Largely Independent of Germline DH Sequence
Source: Front Immunol. 2016 Aug 10;7:296. doi: 10.3389/fimmu.2016.00296 (PMC4979587; doi:10.3389/fimmu.2016.00296)

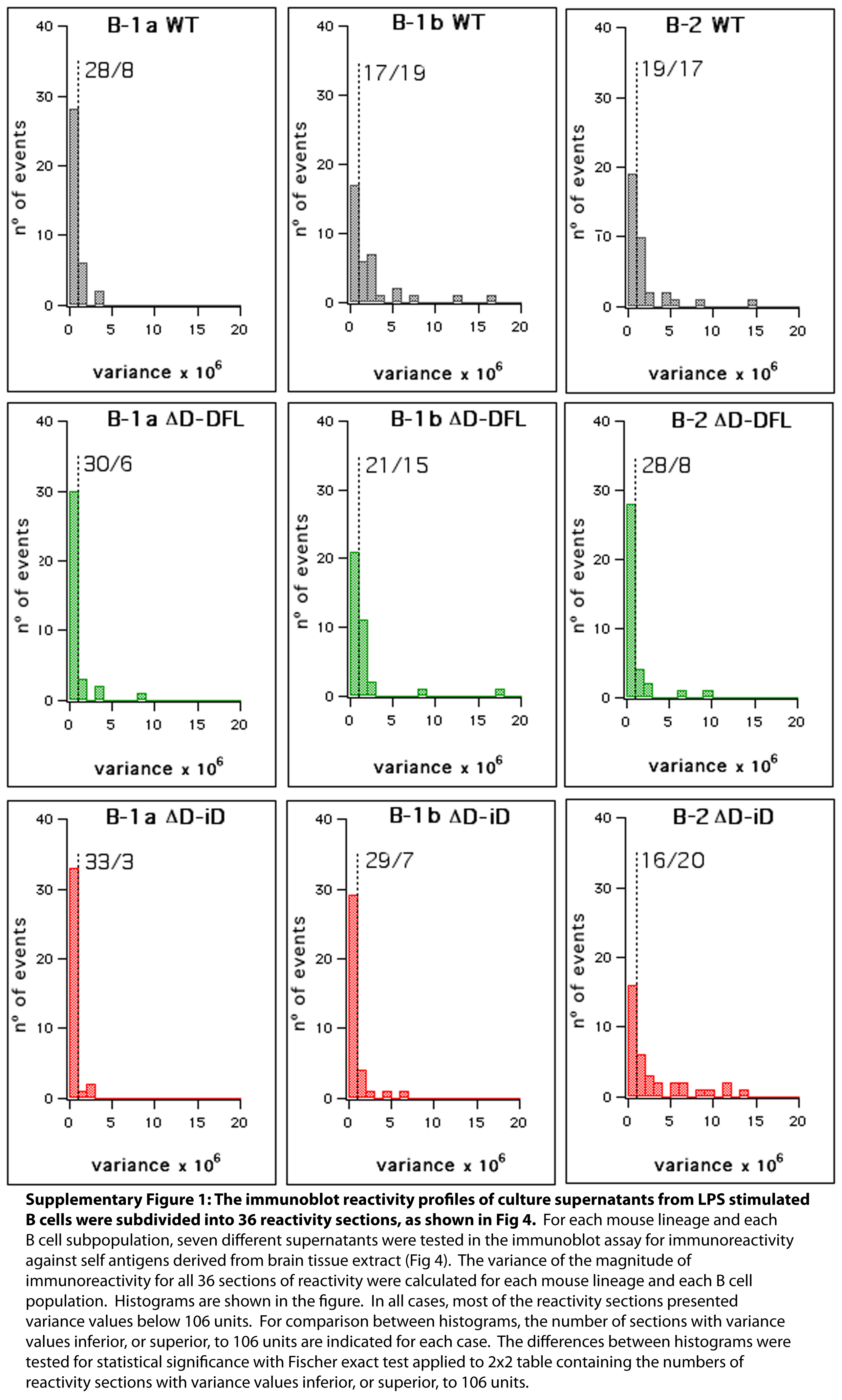

Supplement: Figure S1 — The immunoblot reactivity profiles of culture supernatants from LPS-stimulated B cells were subdivided into 36 reactivity sections, as shown in Figure 4. For each mouse lineage and each B cell subpopulation, seven different supernatants were tested in the immunoblot assay for immunoreactivity against self-antigens derived from brain tissue extract (Figure 4). The variance of the magnitude of immunoreactivity for all 36 sections of reactivity was calculated for each mouse lineage and each B cell population. Histograms are shown in the figure. In all cases, most of the reactivity sections presented variance values below 106 units. For comparison between histograms, the number of sections with variance values inferior, or superior, to 106 units was indicated for each case. The differences between histograms were tested for statistical significance with Fischer exact test applied to 2 × 2 table containing the numbers of reactivity sections with variance values inferior, or superior, to 106 units. [file image_1.tif]
